# Emotional lability secondary to the application of a very potent topical corticosteroid

**DOI:** 10.4103/0019-5545.55093

**Published:** 2009

**Authors:** Srinivasa Sastry Malladi

**Affiliations:** Consultant Psychiatrist, Northamptonshire Healthcare NHS trust, Psychiatry for the elderly, Mason House, Glapthorn Road, Oundle PE8 4JA, UK

**Keywords:** Topical corticosteroids, emotional lability, clobetasol

## Abstract

A 66 yrs old gentleman presented with severe mood changes following application of very potent topical corticosteroid cream, clobetasol propionate, which was prescribed for his recalcitrant eczema. The symptoms disappeared within 24 hours of discontinuation of the cream and he remained mentally well when reviewed after 2 weeks.

## INTRODUCTION

This case report highlights the rare manifestation of psychiatric problems secondary to topical corticosteroids. Having performed thorough literature search I would like to discuss in this report the evidence for this relation and stress the importance of appropriate usage of topical corticosteroids.

## CASE REPORT

A 66 yrs old gentleman was referred for urgent psychiatric assessment as he complained of severe mood swings, which ranged from anger to tearfulness. He had no previous psychiatric history. When seen he gave a 2-week history of exaggerated egosyntonic unipolar affective reactions with symptoms of overwhelming tiredness and sadness, and difficulty in concentrating. A few months earlier, he was diagnosed with eczema in a dermatology clinic and was prescribed fucibet cream (betamethasone with fusidic acid), which is classed as potent corticosteroid. His skin condition did not improve satisfactorily and was considered to have recalcitrant eczema. He was then prescribed clobetasol propionate cream, which is classed as a very potent corticosteroid. As advised, he applied the cream twice daily and to the areas affected i.e. forehead, neck, arms, and chest. Ever since he commenced clobetasol cream, he began to experience severe mood swings. He described these experiences to immediately follow the application of the cream to his body and the episodes lasted upto couple of hours each time. Despite these experiences, he continued to apply the cream, as he has seen an improvement with his skin condition. His medication history suggests that he has drug sensitivity as he reacted badly to local anesthetic for a nasal surgery and he is also allergic to penicillin.

He is not known to drink alcohol, smoke cigarettes or take illicit substances. He was not on any other medications at the time of contact and had no significant medical history. He denied any other difficulties in his life and there were no other symptoms of depression, mania, anxiety, psychosis, or cognitive impairment. There is no family history of psychiatric illness.

From the assessment, given the clear relation between the commencement of the corticosteroid cream and the affective reactions and the absence of any other significant contributors to the presentation, it was concluded that the psychiatric manifestation was secondary to the topical corticosteroid preparation, clobetasol propionate. He was advised to withhold it with a plan to review him in few days time. When reviewed he reported a dramatic improvement within 24 hours of discontinuing the cream and he no longer experienced the symptoms described. He was reviewed again in 2 weeks time when he remained mentally well.

## DISCUSSION

Clobetasol is indicated in the British National Formulary for treatment of severe resistant inflammatory skin disorders such as recalcitrant eczema unresponsive to less potent corticosteroids.[1] Topical corticosteroids are classified according to their potency into mild, moderate, potent, and very potent categories.[1] Clobetasol and fucibet are classed as very potent and potent corticosteroids, respectively (1). Unwanted effects are directly related to the potency of the topical corticosteroids.[2] It is recognized that topical corticosteroid preparations can be absorbed through the skin and may result in suppression of hypothalamo–pituitary–adrenal (HPA) axis and may cause Cushing's syndrome.[2]

Patients with Cushing's syndrome may suffer with psychiatric disturbance including emotional lability, euphoria, depression, psychosis, or mania.[3] Behavioral disturbance is also a recognized feature with difficulty in concentrating, agitation, and reduced energy.[3] These features were clearly described by this patient as consistent features. According to Kaplan and Sadock's synopsis of psychiatry, administration of high doses of exogenous corticosteroids typically leads to a secondary mood disorder similar to mania, and severe depression may follow the termination of steroids.[4] The mechanism by which corticosteroids affect behavior is likely to be multifactorial. Corticosteroid receptors located in areas of the brain such as hippocampus, septum, and amygdala are related to affective and behavioral changes and are likely to affect this through an interaction with monoamine neurotransmitters.[5] While it is possible that oral corticosteroids and possibly topical preparations may result in mood changes, we are not aware of any such previous case reports.

Inhibition of the HPA axis by an excessive application of moderately potent or relatively modest doses of stronger steroids is well documented.[6] Hence, it is important to ensure that the patient applies the cream strictly as advised by their doctor. The weekly application of the clobetasol cream should not exceed 50 g in order to avoid suppression of the HPA axis.[7] The results from the study by Allenby *et al*. also suggest that using less than 50 g of clobetasol propionate ointment a week may result in transient suppression of HPA function, which apparently recovers as the skin heals. This is probably because less ointment is applied and the epidermal barrier is restored, thereby reducing corticosteroid absorption.[8] It is also important to restrict the application of the cream to only the affected areas, as it appears that the widespread application of clobetasol propionate may be equivalent to systemic steroid administration in its effects on the pituitary–adrenal axis.[9]
